# Study on the Synthesis of Chabazite Zeolites via Interzeolite Conversion of Faujasites

**DOI:** 10.1155/2021/5554568

**Published:** 2021-03-29

**Authors:** Long Van Dang, Thu Thi Minh Nguyen, Dang Van Do, Son Thanh Le, Trong Dinh Pham, Anh Thi Mai Le

**Affiliations:** VNU-University of Science, Vietnam National University, Hoan Kiem, Hanoi 100000, Vietnam

## Abstract

The interzeolite conversion of faujasite (FAU-type) zeolites to chabazite (CHA-type) zeolite in the presence of N,N,N-trimethyladamantammonium and N,N,N-dimethylethylcyclohexylammonium cations was investigated over a large compositional range by carefully controlling the reaction mixture compositions. Highly crystalline CHA zeolites were also obtained by the transformation of several zeolite types including EMT, LTL, LEV, RTH, and MFI frameworks. The formation of CHA zeolite from FAU zeolite precursors was substantially faster than that from zeolite L with a similar composition. High-silica CHA zeolites were also produced successfully using a mixture of TMAda with a number of less expensive organic structure-directing agents. The CHA zeolite materials have been synthesized with high crystallinity and with a Si/Al ratio ranging from 5 to 140. Our data support the importance of structural similarity between the zeolite precursors, nucleation/crystallization processes, and the zeolite product in the interzeolite conversion compared to conventional amorphous aluminosilicate gels. Our synthetic methods could be used to prepare other 8-membered ring zeolites such as AEI and AFX frameworks, potential candidates for selective catalytic reduction of NOx, light olefin production, and CO_2_ abatement.

## 1. Introduction

Crystalline zeolite materials possessing uniform channel systems and molecular‐sized pore windows have been widely used as catalysts and adsorbents in a variety of industrial processes, including petrochemical and fine chemical processes [1–4]. Small pore zeolites possessing 8-membered ring (8MR) window apertures have recently been studied extensively as catalysts for efficiently reducing NOx emission from the exhaust of diesel vehicles [5–7], the methanol-to-olefins [8–10], and adsorbents for gas separations such as CO_2_/N_2_ [11–13], CO_2_/CH_4_ [14, 15], and small hydrocarbon separations [16, 17]. During the past decade, the majority of the NH_3_-SCR (selective catalytic reduction) technology research has been focusing on transition metal-exchanged small pore CHA-type (SSZ-13) zeolite (Cu/Fe.CHA/SSZ-13) due to its high NOx conversion over wider temperature windows, enhanced N_2_ selectivity, improved hydrothermal stability, and high hydrocarbon tolerance compared to medium and large pore zeolite-supported catalysts [18–20]. The CHA-based catalyst was commercialized in 2010 and is currently being widely used for NH_3_-SCR of NOx in vehicle emission control [5, 20, 21]. To meet the continuously stringent regulations on NOx emission in the United States, Europe, and other countries, researchers are constantly searching for improved SCR catalysts [6, 7, 22].

**Figure 1:**
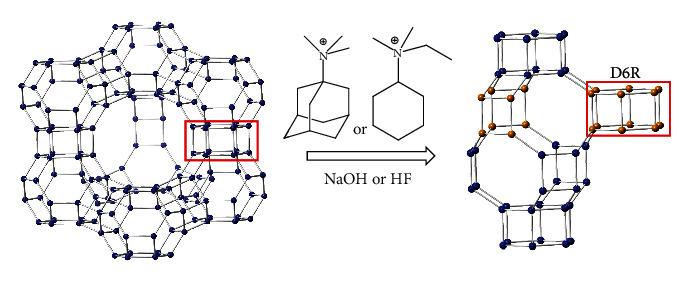
A schematic view of faujasite (left) framework and its transformation into chabazite (right) zeolite under hydroxide and fluoride media. Oxygen atoms were omitted for clarity. The double 6-rings (D6Rs) of faujasite and chabazite zeolites were highlighted.

The development of various synthesis routes has had a significant impact on extending the existing synthesis field for high-silica microporous zeolites [23, 24]. In general, the synthetic zeolites are prepared under hydrothermal conditions from amorphous silica and alumina sources. However, alternative methods of synthesis using zeolites as Si and Al precursors could be used to improve the zeolite properties or the synthesis process. Small pore zeolites such as LEV, CHA, AEI, SFW, ERI, and AFX have been prepared successfully from the hydrothermal transformation of crystalline faujasite (FAU) zeolites having the same secondary building unit of double 6-rings (D6Rs) [25–32]. FAU has a 3-dimensional pore structure (Figure 1) with pores running perpendicular to each other in the *x*, *y*, and *z* planes. The pore apertures of FAU are defined by 12 rings (7.4 Å) with larger cavities of diameter 12 Å. The cavities in the FAU zeolite are surrounded by small sodalite cages connected to each other via D6Rs [33, 34]. CHA zeolite contains 3-dimensional networks with low framework densities (∼15 T atoms/nm^3^) based on D6R building units and ellipsoidal cages with dimensions of 6.7 Å × 11 Å. Chabazite belongs to R3 m (trigonal) space group [35] and consists of three double 6-membered rings (D6Rs), three large ellipsoidal cavities (7 Å × 10 Å), and nine 8-membered ring windows in one unit cell of 36 T-atoms (Figure 1).

In this study, we have synthesized zeolites with the CHA topology by interzeolite transformation of faujasite zeolites over a broad Si/Al range (5 to ∼140) using N,N,N-trimethyladamantammonium (TMAda) and dimethylethylcyclohexylammonium (DMECHA) in hydroxide and fluoride media. We also investigated in the direct transformation of various zeolite framework types FAU, LTL, LEV, MFI, EMT, and RTH into CHA zeolites. The synthesized materials were characterized by powder X-ray diffraction (XRD), scanning electron microscopy (SEM), energy-dispersive X-ray spectroscopy (EDX), ^27^Al and ^29^Si magic angle spinning nuclear magnetic resonance (MAS NMR), and liquid N_2_ physisorption. The synthesis methods provide a fast and reliable alternative to the synthesis of CHA-type zeolites with any desired Si/Al ratio larger than 5.

## 2. Materials and Methods

### 2.1. Organic Structure-Directing Agents (OSDAs)

Tetraethylammonium hydroxide (TEAOH, Alfa Aesar, 35% wt aqueous solution), N,N,N-trimethyladamantammonium hydroxide (TMAdaOH, 25% wt aqueous solution, Sachem), 1-adamantanamine (Sigma, 98%), 18-crown-6 (Sigma, 98% wt), tetramethylammonium hydroxide (25% wt, Alfa), tetrapropylammonium hydroxide (Sachem, 40% wt), dimethyldipropylammonium hydroxide (40% wt, Sachem), and choline hydroxide solution (Sigma, 46% wt) were used. Hydroxide solution (Sigma, 46%) was used as received without further purification.

N,N,N-Dimethylethylcyclohexylammonium (DMECHA) was prepared similarly to a report by Mertens [36]: 72.0 g (0.6 mol) ethyl iodide was added drop-wise to a mixture of 49.0 g (0.5 mol) N,N-dimethylcyclohexylamine and 180 g ethanol at room temperature. The solution was heated to about 70°C overnight. The reaction product was then cooled to room temperature and kept in the refrigerator for 2 hours to crystallize the organic salt. The solid obtained was filtered, washed exhaustively with diethyl ether, and dried in the oven at 70°C for 1 h giving a yellowish powder (∼96% yield). ^1^H NMR (400 MHz, D_2_O): 3.29 (2H), 3.22 (1H), 2.85 (6H), 2.0 (2H), 1.82 (2H), 1.55 (1H), 1.41 (2H), 1.27 (2H), 1.21 (3H), 1.05 (1H). ^13^C NMR (400 MHz, D_2_O): 71.1, 58.1, 47.4, 25.5, 24.9, 24.3, 7.3.

The ion exchange of DMECHA iodide to its hydroxide form was carried out using Amberlyst A26 hydroxide exchange resin. The typical mass ratio employed for the ion exchange was 1:3:5 to the OSDA as halide: Amberlyst resins: water. The exchange was carried out overnight at room temperature. The hydroxide solution was finally evaporated to a desired concentration on a BUCHI Rotavapor.

### 2.2. Zeolite Synthesis

*Reagents.* Reagents were N-sodium silicate solution (∼28 wt% SiO_2_, 8.9 wt% Na_2_O, PQ Corporation), zeolite FAU (CBV100, CBV500, CBV712, CBV720, and CBV780 from Zeolyst), zeolite FAU (320NAA, 341NHA, 350HUA, 360HUA, 385HUA, and 390HUA from Tosoh), NaOH 1 N (diluted from NaOH 99% Fisher Scientific), deionized (DI) water, and HF (Sigma, 48% wt).

#### 2.2.1. Interzeolite Transformation in Hydroxide Media

*Synthesis of Chabazite Using Faujasites (FAUs) as Aluminum Sources*. A typical synthesis was carried out following the literature [27, 37]: 4 mmol of either TMAda/DMECHA hydroxide was mixed with 0.4 g of 1 M NaOH, and the mass was brought to 12 g with water. Next, 5 g of N sodium silicate solution was added, and the gel was stirred for about 5 minutes. Finally, 0.5 g of FAU zeolite (CBV100, CBV500, CBV712, CBV720, 320NAA, 341NHA, 350HUA, or 360HUA) was added as an aluminum source. The reaction was heated at 150°C and rotated at 40 rpm for 5 days. The composition of the synthesis gel was as follows: 26 H_2_O :  0.27 Na_2_O : 1 SiO_2_ : *x* Al_2_O_3_ : 0.14 DMECHA-OH/TMAdaOH (where *x* varies depending on the faujasite sources).

*Synthesis of Chabazite Using Other Zeolites (EMT, LEV, LTL, and RTH) as Aluminum Sources*. The following list describes the synthesis of the materials used as a source of aluminum:

- LTL zeolite (HSZ-500, Si/Al = 3) and MFI zeolite (CBV2314, Si/Al = 11.5) were purchased from Tosoh and Zeolyst, respectively.
- *Synthesis of LEV.* LEV-type zeolite with Si/Al = 15 was synthesized from CBV720 (Si/Al = 15) FAU-type zeolite using 1-adamantanamine (Ada-NH_2_) as an OSDA following a similar procedure described in the report by Shibata et al. [28]. The mixture with molar compositions of H_2_O/Ada-NH_2_/SiO_2_/Al/NH_4_F = 10/0.3/1/0.067/0.05 was placed in an oven at 150°C/7 days under rotation at 40 rpm.
- *Synthesis of EMT.* EMT-type zeolite with Si/Al = 3.5 was synthesized using 18-crown-6 as an OSDA and NaF as a mineralizer with a gel composition of 10 SiO_2_ : Al_2_O_3_ : 1.1 Na_2_O : 1.1 NaF : 18-Crown-6 : 140 H_2_O according to the procedure described by J. Berger et al. [38]. The gel was stirred at room temperature for 1 day and then at 110°C for 8 more days.
- *Synthesis of RTH.* RTH-type zeolite with Si/Al = 10 was synthesized in a fluoride medium. The OSDA pentamethylimidazolium and RTH zeolite were synthesized following a similar procedure described by Schmidt et al. [39]. The final molar ratio of the gel SiO_2_/Al/ROH/HF/H_2_O = 1/0.067/0.5/0.5/7 was placed in an oven at 160°C/10 days under rotation with a speed of 60 rpm.
- Prior to their use as Al source for CHA synthesis, all as-made materials (LEV, EMT, and RTH) were calcined at 580°C by heating at a rate of 3°C/min for 8 hours.

*Chabazite zeolites* were prepared by a procedure similar to the one using faujasite as aluminum source, except that faujasite was replaced by either of the following zeolites (LTL, MFI, LEV, EMT, or RTH) as an aluminum source, and the gel mixture was heated at 150°C and 40 rpm for 7 days instead of 5 days.

#### 2.2.2. Interzeolite Transformation in Fluoride Media

The procedure for the synthesis of CHA zeolites in fluoride media was modified from the literature [40–42]. Typically, faujasite zeolites as both silica and aluminum sources were mixed with TMAdaOH solution and heated to about 50–60°C to remove the water. Then HF 48% wt was added to the mixture to produce the final gel compositions of 3 H_2_O : 1 SiO_2_ : *x* Al_2_O_3_ : 0.5 TMAdaOH : 0.5 HF. This thick paste was homogenized in a Teflon container manually and transferred to a 23 mL Teflon-lined stainless steel autoclave (Parr). The autoclave was kept at 150°C under rotation for 5 days in a convection oven.

*Calcination.* All as-made chabazite products were calcined in a forced-air furnace. The materials were heated to 120°C at a rate of 3°C/min, held for 2 h, then heated to 580°C at a rate of 3°C/min, and held for 8 h to ensure complete combustion of the organics.

### 2.3. Analytical Section

The scanning electron microscopy (SEM) images and energy-dispersive X-ray spectroscopy (EDS) of the synthesized zeolites were obtained using Auriga 60 CrossBeam (FIB/FE-SEM) microscopes, operating at an acceleration voltage of 1.5–3 keV and a current of 10 *μ*A.

The X-ray powder diffraction (XRD) patterns were collected at room temperature on a Philips X'Pert Panalytical powder diffractometer using Cu K*α* radiation (*λ* = 1.5418 Å). The data were collected in a stepwise fashion of 2*θ* ranging from 5.0° to 40.0° with a step size of 0.02° and 2 s per step.

The micropore volume and surface area of the zeolite samples were measured using N_2_ adsorption isotherms at 77 K with a Micromeritics ASAP 2020 device. Before the adsorption measurements, each sample was degassed at a temperature of 350°C for 6 h. The specific surface area (*S*_BET_) and microporous volume (*V*_micro_) were calculated using the BET and t-plot methods, respectively.

^29^Si and ^27^Al magic angle spinning nuclear magnetic resonance (MAS NMR) spectra were recorded on a Bruker AVIII-500 solid-state NMR spectrometer and a Bruker 4 mm MAS probe. The spectral operating frequencies were 500.1 MHz, 130.3 MHz, and 99.3 MHz for ^1^H, ^27^Al, and ^29^Si nuclei, respectively.

## 3. Results and Discussion

### 3.1. Interzeolite Conversion in Hydroxide Media

Faujasite-type zeolites have been used as a catalyst in an industrial process for a long time due to their high activity and low cost [43, 44]. Moreover, FAU can be used as an aluminum source for variety of zeolites' synthesis such as ZSM-5, ZSM-11 [45], beta, SSZ-37 [46], STF, MTW, chabazite [27, 47], and LEV [28]. In this work, we used various faujasite sources (different Si/Al ratios) as aluminum reagents for chabazite synthesis. The transformation of FAU into CHA zeolite under similar synthesis conditions using TMAda as the OSDA has been reported [27, 48]. Herein, we focused on the use of DMECHA as the OSDA to successfully synthesize CHA zeolites with various Si/Al ratios (Table 1).

**Figure 2:**
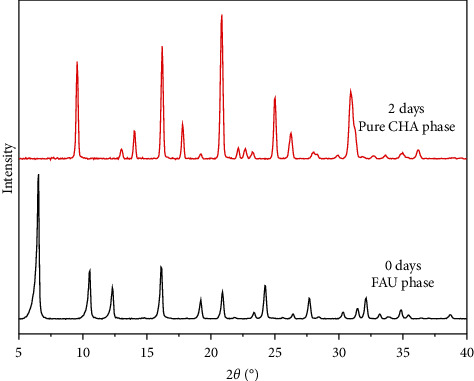
X-ray diffraction patterns of faujasite (0-day crystallization) and CHA (2-day crystallization) using CBV100 as Al source and DMECHA as the OSDA. Pure CHA phase obtained after 2 days indicated complete transformation of FAU precursors.

**Figure 3:**
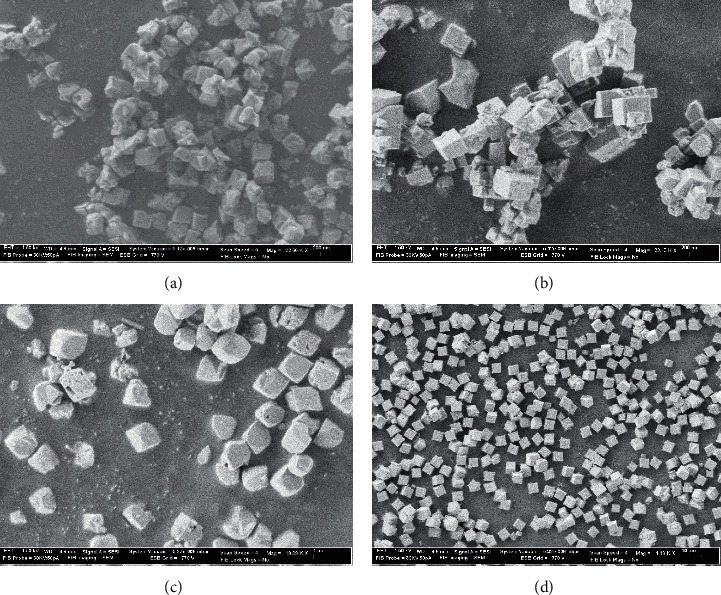
SEM images of CHA zeolites prepared by the transformation of CBV100 ((a), sample #1), CBV712 ((b), sample #3), MFI ((c), sample #11), and LTL ((d), sample #13).

**Figure 4:**
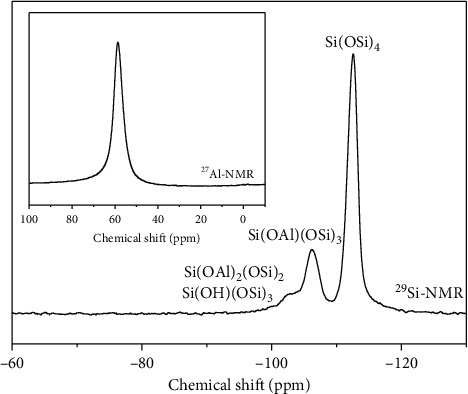
^29^Si single pulse and ^27^Al NMR of calcined CHA zeolite (Si/Al = 10.8, sample #3).

X-ray pattern (Figure 2) indicated a complete transformation of faujasite into CHA phase in less than two days. The synthesized chabazites have rhombohedral (pseudocubic) morphology with a particle size of about 0.5–1 *μ*m (Figure 3).  Figure 4 shows the ^29^Si and ^27^Al NMR spectra of sample #3. The ^27^Al MAS NMR spectrum of the calcined CHA zeolite #3 showed only one peak at approximately 58 ppm, corresponding to the tetrahedrally coordinated framework aluminum species. No peak corresponding to octahedrally coordinated aluminum (extraframework aluminum species) was observed at around 0 ppm. The NMR resonance at −113 ppm was assigned to Q^4^(0Al) or Si(4Si) configuration of the one T site of the three samples. The weak band at around −106 ppm reflects Q^4^(1Al) or Si(3Si, 1Al), and the broad band at −103 ppm represents either Q^4^(2Al) or Q^3^(0Al) (silanol groups Si(3Si, OH)) atoms. The results of our cross-polarization experiment (not shown) indicate that the signal at about −106 ppm corresponds to the Q^4^(1Al) atoms, while the band at around −103 ppm relates to Q^3^(0Al) species. The silicon to framework aluminum ratio of the sample (Si/Al = 10.8 by EDX, much lower than Si/Al = 30 as determined by NMR if we consider that the peak at −103 ppm belongs to Q^3^(0Al) species) indicates that the band around −103 ppm corresponds to both Q^3^(0Al) and Q^4^(2Al) species (Figure 4).

We observed that the mass and Si/Al ratios in the chabazite products approximately double over the aluminosilicate reactants (FAUs with Si/Al ratios <7). This indicated that almost all of Al in the faujasite sources and part of Si in sodium silicate source were incorporated into the final chabazite products. The Si/Al ratios in the chabazite products are close to Si/Al ratios in gels when FAUs with Si/Al ratios of >12 were used, thus demonstrating the incorporation of Al and Si of only FAU reactants into the chabazite products. In fact, we are able to produce CHA (Si/Al = 15) from FAU (Si/Al = 15) without the presence of any other Si or Al resources with the gel composition of 25H_2_O : 0.07NaOH : 1SiO_2_ : 0.033Al_2_O_3_ : 0.3 (DMECHA/TMAda). When higher Si/Al of faujasite was used (CBV760, Si/Al = 30), amorphous silica colloid particles were formed.

**Figure 5:**
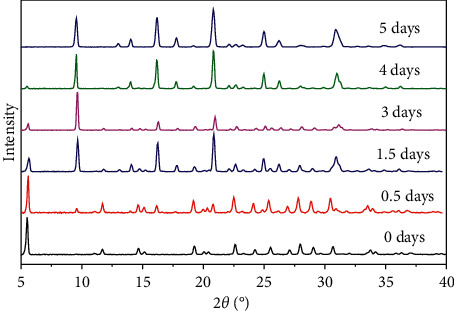
X-ray diffraction patterns of as-made zeolite products (0 to 5 days) prepared using LTL as Al source and DMACHA as the OSDA.

Compared with the transformation of Y zeolites (Figure 2), zeolite L (LTL-framework type) transformation into chabazite zeolite at the same hydrothermal condition (150°C) took longer. As seen in Figure 5, the dissolution of zeolite L and the growth of CHA co-occur, and that undissolved zeolite L still exists in the solid mixtures up to 5 days under the synthesis conditions. Experimental and theoretical studies have suggested that the stability of zeolites decreases as the zeolite framework density decreases [49, 50]. Considering that FAU has a lower framework density (13.3) than LTL (16.7), this result is consistent with the lower stability of FAU under hydrothermal synthesis conditions. In addition, FAU and CHA-type zeolites contain similar secondary building units (only D6Rs) compared to LTL (D6Rs, 6MR, and 4MR), and the nucleation and growth of CHA should occur more readily from the partial dissolution of FAU zeolite than LTL-type zeolite.

EMT has a framework structure related to FAU and the same FD (13.3) and composite building units (D6R, SOD cage); thus it could be used as a replacement for FAU zeolite as an Al source for the synthesis of chabazite zeolites (Table 2). To continue, we also investigated on the transformation of more dense 8MR zeolites (RTH, FD = 16.1, and LEV, FD = 15.9), 10MR zeolite (MFI, FD = 18.4), and 12MR zeolites (BEA, MOR, FD = 15.3 and 17, respectively) to less dense CHA zeolite (Table 2). The results indicated that RTH, LEV, and MFI were easily dissolved and transformed into zeolite CHA under hydrothermal treatment with the presence of OSDA TMAda. This implies that in all those cases host-guest interactions in the less dense phase (CHA zeolite) are strong enough to overcome the energy penalty associated with the change from denser phases to a more porous framework. The transformation of a dense phase to a less dense phase has also been previously reported [51–53]. A more effortless interzeolite transformation of RTH and LEV is due to their possessing 4-rings and 6-rings as CHA zeolite. MFI, BEA, and MOR zeolites contain mostly 5-rings and fewer 4-rings; however, only MFI was able to convert to CHA product. The occlusion of TMAda cations inside the pore channels of large pore zeolites by the ion-exchange or adsorption could stabilize these BEA and MOR zeolites under the hydrothermal synthesis conditions and thus prevent these zeolites from transforming into CHA zeolites. Our observation is consistent with a study by Zones, in which the introduction of a smaller organocation could inhibit the rate of conversion of FAU to CHA [27].

**Figure 6:**
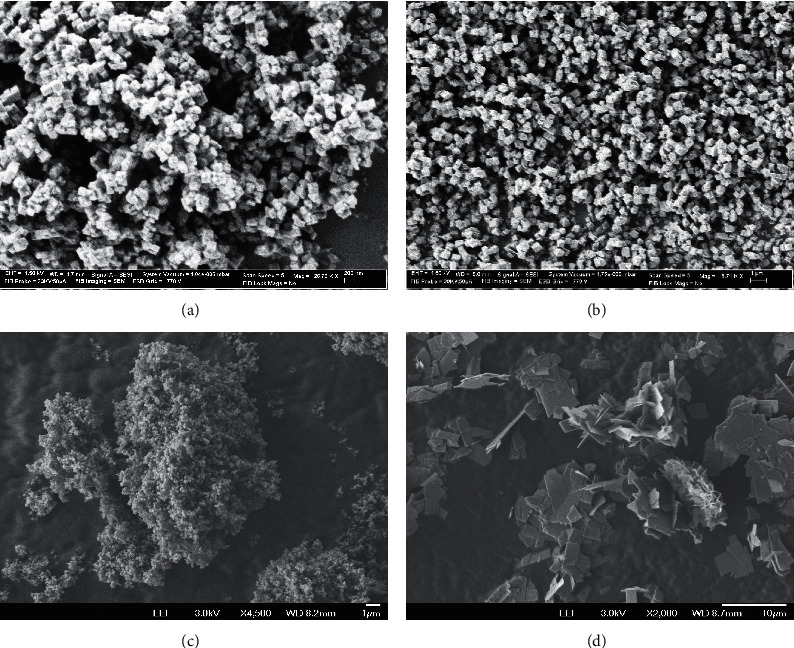
SEM images of the zeolite product samples #18 (a), #20 (b) #27 (c), and #32 (d).

We pursued a further investigation into the synthesis of chabazite zeolites (Si/Al ≈ 10) using various OSDAs in companion with TMAda (Table 3). The chabazites prepared using mixed templates consist of pseudocubic crystals with a uniform particle size of 300–500 nm (Figures 6(a) and 6(b)). As long as the second OSDA is not strong enough to direct for the formation of other zeolite phases, CHA phase will be produced in the presence of two OSDAs, as shown in Table 3 and our previous study on pure silica chabazite [41].

### 3.2. Interzeolite Conversion in Fluoride Media

In our previous study, high-silica CHA zeolites were prepared in fluoride media using conventional Si (TEOS) and Al (Al(OH)_3_ & Al(O-iPr)_3_) precursors. Moteki and Lobo [40] used low silica zeolites such as CBV500 as Al source, in combination with TEOS as Si source to synthesize various high-silica zeolites including LTA, ITW, STT, CHA, and BEA. Herein, high-silica faujasites were solely used as both Si and Al precursors in their transformation into CHA zeolites. The results in Table 4 indicated that faujasites with Si/Al ≤ 6 did not yield a pure phase of CHA zeolite. Higher Si/Al ratios of the zeolite precursors were required for a successful transformation. The results are consistent with a theoretical value of 3OSDA/unit cell of CHA containing 36 T-atoms or a minimum Si/Al ratio of 11 [41, 54] in the final CHA products synthesized by the fluoride route. At a high Si/Al ratio (Si/Al = 150) and with an increase in the water content (H_2_O/SiO_2_ = 3–15), the product gradually changes the phase selectivity of the crystallization from less dense phase CHA to a mixture of CHA and STT and a denser phase STT zeolite. A dense framework structure usually requires a smaller concentration of OSDA cations, which is commonly observed with low heteroatom substitutions and more diluted synthesis conditions [55–57].

The dissolution of faujasite zeolites produced 4MRs and D6Rs building units, which led to the quick formation of high amount of nuclei. This resulted in the formation of smaller crystals (<200 nm, Figure 6(c)) as compared to CHA prepared by using conventional Si and Al precursors (1–5 microns) [41, 58].

**Figure 7:**
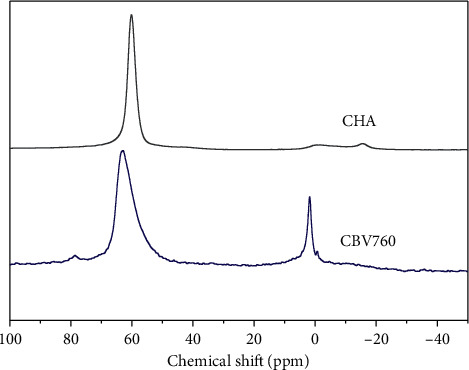
^27^Al MAS NMR of faujasite CBV760 and CHA sample #27.

High-silica FAU zeolites such as CBV760 were obtained by a dealumination method; thus a decent amount of extraframework Al (20.6%) was expected on CBV760 sample (Figure 7, *δ*   0 ppm). The relative peak intensities of ^27^Al MAS NMR spectrum (Figure 7) of sample #27 indicated that the material contains mostly framework Al (*δ*   60 ppm) with minimal amount (≈3%) of extraframework Al (*δ*   0 ppm). The low extraframework Al in the final product indicated that extraframework Al in the FAU precursor could be dissolved in HF and incorporated into the CHA product framework.

## 4. Conclusions

High-silica CHA zeolites with various Si/Al ratios can be modified by using different faujasite sources in both hydroxide and fluoride media, and a variety of CHA morphologies can be prepared by interzeolite transformation of several types of zeolites such as FAU, EMT, LEV, RTH, MFI, and LTL frameworks. The comparative study of the transformation of two zeolites showed that zeolite faujasite is a more efficient precursor for the synthesis of CHA than zeolite L. The growth rate of CHA yielded from zeolite FAU is faster, which is attributed to the significant similarities of FAU-type and CHA-type structures and the lower framework density of the FAU zeolite. The use of faujasite possessing similar structure building units 4MRs, 6MRs, and D6Rs as CHA zeolite in the synthesis gel appears to be crucial in order to obtain CHA phase. The nucleation/crystallization processes of interzeolite transformation method can be modified compared to conventional amorphous aluminosilicate gels, which led to the formation of various morphology and particle size. In summary, this study provides a facile and rapid way for the synthesis of CHA zeolite, an industrially relevant zeolite with great impact on environmental protection and light olefins synthesis. The combination of a low-cost template, easy synthesis routes, and the ability to produce a wide range of Si/Al ratio CHA products makes DMECHA an attractive OSDA for broad applications of CHA zeolite in catalysis and adsorption.

## Figures and Tables

**Table 1:** Synthesis parameters of high-silica CHA zeolites using faujasites as Al sources and some product properties.

Sample no.	Synthesis conditions		Product		
	Al source	Si/Al faujasite	Phase	Si/Al	*V*_mic_ (cm^3^/g)
1	CBV100	2.5	CHA	5.2	—
2^*∗*^	CBV500	2.5	CHA	5.9	0.26
3^*∗*^	CBV712	6.0	CHA	10.8	0.27
4^*∗*^	CBV720	15.0	CHA	14.7	0.27
5	CBV760	30.0	Amor	—	—
6	320NAA	2.7	CHA	6.1	—
7	341NHA	3.5	CHA	8.6	—
8	350HUA	5.0	CHA	11.4	0.27
9	360HUA	7.5	CHA	12.1	—

Gel compositions: 26 H_2_O : 0.27 Na_2_O : 1 SiO_2_ : *x* Al_2_O_3_ : 0.14 DMECHA-OH. ^*∗*^TMAdaOH was also used in addition to DMECHA. Amor: amorphous phase. —: did not measure.

**Table 2:** Synthesis parameters of high-silica CHA zeolites by interzeolite transformation and some product properties.

Sample no.	Synthesis conditions			Product		
	OSDA	Zeolite	Si/Al_Zeolite_	Phase	Si/Al	*V*_mic_ (cm^3^/g)
10	TMAda	BEA25	12.5	BEA	—	—
11	TMAda	MFI23	11.5	CHA	22.1	—
12	TMAda	MOR13	6.0	MOR	—	—
13	TMAda^*∗*^	LTL	3.0	CHA	6.1	0.25
14	TMAda^*∗*^	LEV	14.7	CHA	15.3	—
15	TMAda^*∗*^	EMT	3.5	CHA	7.2	—
16	TMAda^*∗*^	RTH	5.0	CHA	8.3	—

^*∗*^DMECHA was also used in addition to TMAda.

**Table 3:** Synthesis of CHA zeolites using a mixture of two organic templates.

Sample no.	Synthesis conditions			Product	
	Si/Al	OSDA1	OSDA2	Phase	Bulk Si/Al
17	57	TMAda	DMDPA	CHA + imp	—
18	57	TMAda	TEA	CHA	10.5
19	57	TMAda	TPA	CHA	10.7
20	57	TMAda	TMA	CHA	—
21	57	TMAda	Ada-NH_2_	CHA	—
22	57	TMAda	Choline	CHA	10.1

Gel compositions: 26 H_2_O : 0.27 Na_2_O : 1 SiO_2_ : 0.017 Al_2_O_3_ : 0.07 TMAda : 0.07 OSDA2. —: did not measure; imp: unknown impurities.

**Table 4:** Synthesis parameters of high-silica CHA zeolites by interzeolite transformation in fluoride media and some product properties.

Sample no.	Synthesis conditions			Product		
	Si, Al source	Si/Al	H_2_O/SiO_2_	Phase	Si/Al	*V*_mic_ (cm^3^/g)
23	CBV500	2.5	3	FAU	—	—
24	CBV712	6.0	3	CHA + FAU	—	—
25^*∗*^	CBV720	15.0	3	CHA	14.7	0.26
26	CBV720 + CBV760	22.0	3	CHA	21.1	—
27^*∗*^	CBV760	30.0	3	CHA	28.1	0.27
29	HUA385	50.0	3	CHA	45.3	—
30	HUA390	150.0	3	CHA	136.9	0.27
31	HUA390	150.0	10	STT + CHA	—	—
32	HUA390	150.0	15	STT	—	0.20

^*∗*^DMECHA was also used in addition to TMAda, and the rest of the CHA samples were prepared using TMAda as the OSDA.

## Data Availability

The data used to support the findings of this study are included within the article.
